# Legacy Effects of Climate Extremes in Alpine Grassland

**DOI:** 10.3389/fpls.2018.01586

**Published:** 2018-10-30

**Authors:** Hans J. De Boeck, Erika Hiltbrunner, Maya Verlinden, Seraina Bassin, Michaela Zeiter

**Affiliations:** ^1^Centre of Excellence PLECO (Plants and Ecosystems), Department of Biology, Universiteit Antwerpen (Campus Drie Eiken), Wilrijk, Belgium; ^2^Institute of Botany, Department of Environmental Sciences, University of Basel, Basel, Switzerland; ^3^Agroscope, Climate and Agriculture Group, Zurich, Switzerland; ^4^School of Agricultural, Forest and Food Sciences, Bern University of Applied Sciences, Zollikofen, Switzerland; ^5^Institute of Plant Sciences, University of Bern, Bern, Switzerland

**Keywords:** biomass, drought, heat wave, mountain, recovery, resistance, stress, warming

## Abstract

Climate change is particularly apparent in many mountainous regions, with warming rates of more than twice the global average being reported for the European Alps. As a result, the probability of climate extremes has increased and is expected to rise further. In an earlier study, we looked into immediate impacts of experimentally imposed heat waves in alpine grassland, and found that these systems were able to cope with heat as long as enough water was available. However, concomitant drought led to increased stress, and reduced aboveground biomass production and green plant cover. Here, we studied the legacy effects (lag-effects) of the imposed climate extreme to see whether delayed responses occurred and how fast the alpine grassland could rebound from the initial changes. Green cover continued to be suppressed the two following years in communities that had been exposed to the most intense hot drought, while aboveground biomass production had returned to control levels by year 2. The initial lower resistance of the forb fraction in the communities was not compensated by faster recovery later on. This resulted in alpine communities that became (and remained) relatively enriched with graminoids, which resisted the original extreme better. The responses of alpine grassland to heat extremes with or without drought observed in this study resemble those typically found in lowland grassland in the short term. However, alpine grassland exhibited longer legacy effects from an annual perspective, with delayed recovery of aboveground production and persistent changes in community composition. This suggests that once initial resistance thresholds are exceeded, impacts may be longer-lasting in alpine grassland, where recovery is constrained by both the short growing season and difficult seedling establishment.

## Introduction

Ongoing climate change is characterized by a relatively modest rise in global surface temperatures to date, yet this has led to a much more pronounced increase in the number of heat waves. The current 0.85 K warming has made preindustrial 0.1-percentile heat extremes ~5 times more likely (Fischer and Knutti, 2015). With continued anthropogenic warming (IPCC, 2013), the number of heat waves will increase further, making current extreme events common occurrences by the second half of the twenty-first century (Christidis et al., 2015). Moreover, concurrent heat and drought extremes are thought to occur more frequently than their single likelihoods would suggest. Indeed, as the water and energy balance are entwined, temperatures tend to rise during drought as energy dissipation shifts from latent to sensible heat (De Boeck and Verbeeck, 2011), while the anticyclonic conditions associated with most heat waves result in gradual drying through low precipitation and high atmospheric demand for water vapor (Trenberth and Shea, 2005; De Boeck et al., 2010).

Climate extremes can profoundly impact plant communities and ecosystems. The aforementioned heat and drought can result in reduced primary productivity (Zscheischler et al., 2014), altered community composition (Hoover et al., 2014), and even system state shifts (Fensham et al., 2009), with effects of the combination of heat and drought generally exceeding the sum of their individual impacts (De Boeck et al., 2011). Some of these effects manifest in the short term, but delayed responses that affect ecosystems after the end of the initial extreme (legacy effects) are also often observed (e.g., Sala et al., 2012; Anderegg et al., 2015). The most pronounced legacy effects are usually found where the initial impacts were highest (Yahdjian and Sala, 2006), but it is also possible that effects of the extreme are small or insignificant in the short term and become apparent only later (Vanoni et al., 2016). Determining how long antecedent conditions can affect plant and ecosystem responses remains challenging. Using a modeling approach, Ogle and co-workers found that antecedent effects explained between 18 and 28% of the response variables in semi-arid and arid ecosystems (Ogle et al., 2015).

The rapid increase in frequency and severity of climate extremes together with the high-impact potential has led to increased scientific interest. Nevertheless, many regions are underrepresented in extreme event studies (Beier et al., 2012), among which the cold biomes with their short growing season. This contrasts with observations of rapid climate change in these biomes, with warming rates significantly exceeding those recorded globally (Gobiet et al., 2014; Overland et al., 2017). At the same time, droughts are expected to become more frequent, longer and more intense the coming decades, also in mountain areas (Calanca, 2007; Gobiet et al., 2014), although regional differences are likely (Beniston et al., 2018). In addition, most extreme event studies focus on responses in the short term (initial resistance and within-season recovery), while reports on longer term impacts, including legacy effects, are less common (Knapp et al., 2012).

In an earlier extensive experimental study on heat and drought extremes in alpine grassland, we found that exposure to heat waves of variable intensity provoked little direct response unless the heat coincided with drought (De Boeck et al., 2016). Both F_v_/F_m_ (a non-invasive measurement of photosystem II efficiency used as a general stress indicator) and canopy greenness decreased linearly with increasing average vapor pressure deficit (a combined measure of temperature and dryness of the air) under drought, resulting in lower aboveground biomass (−40% to −75%). Graminoids initially seemed more resistant than forbs, a trend also observed by Cremonese et al. (2017) following a natural hot drought. Although community-scale green cover was reduced dramatically (−80%) at the hottest site under drought conditions, it was unclear if mortality was widespread or if it was mainly a phenological response that prevented further water loss. In the latter case, this would allow plants to rebound the following growing season, in particular as the majority of alpine plant species are perennials.

In the current study, we focus on the legacy effects of the imposed heat and/or drought. While recovery after climate extremes is often fast in temperate grassland (Dreesen et al., 2014; Griffin-Nolan et al., 2018), the short growing season in the (sub)alpine zone is likely to constrain immediate recovery (De Boeck et al., 2016; Cremonese et al., 2017). This means that, although short-term responses to heat and drought, both as single factors and in combination, were found to be similar in alpine and temperate grassland, the different recovery dynamics may lead to divergent responses in the longer term. However, research on the legacy effects of climate extremes in cold biomes has been scant thus far. Here, we discuss these longer-term responses by reporting on changes in plant cover, aboveground phytomass and community composition during the 2 years (2014 and 2015) following the imposed extremes (2013) in alpine grassland. We hypothesize that (i) decreases in green cover during the extremes were caused by mortality rather than phenological responses, i.e., lower cover should also be apparent the following year; (ii) recovery is slow and legacy effects are predominantly negative; and (iii) the initially more resistant graminoids are able to maintain their increased relative success (De Boeck et al., 2016) also the following years, whereas forb biomass and species richness remains suppressed.

## Materials and methods

### Location and set-up

This experiment is a follow-up from an earlier study (De Boeck et al., 2016), in which swards of alpine grassland including their main rooting horizons (monoliths) were transplanted along an extended (c. 1,800 m) elevation gradient. Monoliths of comparable species composition were collected at the ALPFOR research station, situated at 2,440 m near the Furka pass in the Swiss central Alps (46°34′N 8°25′E). These 590 cm^2^ monoliths were selected on the basis of the presence of five species (the graminoids *Nardus stricta* and *Carex curvula*, and the forbs *Homogyne alpina, Potentilla aurea* and *Geum montanum*) and similarity in cover, to avoid bias between treatments due to differences in community composition. After excavation (early July 2013) the monoliths were transported to three lower-elevation sites: Oberwald (1,390 m a.s.l., 46°32′N 8°21′E), Bister (1,040 m a.s.l., 46°21′N 8°04′E), and Visp (660 m a.s.l., 46°17′N 7°53′E). One batch of monoliths was installed at the site of origin (Furka) as a reference (control). Half of the 48 monoliths was regularly irrigated, the other half was covered by a rainout shelter and was not irrigated. The climate extreme lasted 17 days (15 July-1 August 2013), after which all monoliths were transported back to the reference site, were immediately irrigated and reinstalled into the soil on their original locations on August 19 after aboveground phytomass harvest. A more elaborate description can be found in De Boeck et al. (2016).

### Measurements

#### Meteorology

Meteorological measurements were made by an automatic weather station at the Furka site (operated by ALPFOR) that contributes to the network of the Swiss Federal Office of Meteorology and Climatology (MeteoSwiss). These include air and relative humidity (RH; Rotronic MP102H and Hygroclip HC2-S3, Switzerland) incoming and reflected global radiation (CM11 pyranometer, Kipp & Zonen, The Netherlands), precipitation (Lambrecht 1518H3, Germany), soil temperature (−10 cm, −25 cm; T107 probe Campbell Scientific, USA) and volumetric soil water content (−5, −15 cm; CS616 water content reflectometer, Campbell Scientific, USA).

#### Plant and community responses

Measurements were made following the same protocols used in the first study (De Boeck et al., 2016). The percentage of total green cover (between 0 and 100%), used as an indicator of leaf growth, expansion and senescence, was visually estimated for every monolith by the same observer and without looking at previously recorded data to avoid bias. Standing plant matter (phytomass) was clipped at ~2 cm height, separated per functional group (graminoids, herbs and (one) nitrogen fixer) and into living (green; biomass) and dead (brown; necromass), dried at 70°C for 2 days and then weighed. Cover estimates and phytomass harvests were carried out around the peak of the growing season, and were completed in the first week of August 2014 and 2015, which is ~1 week earlier than in 2013. This was done to reflect the earlier vegetation development due to earlier snow melt in 2014 and 2015 compared to 2013, i.e., to ensure that measures were comparable between years. To complement data on changes in the contribution of functional groups to phytomass, we assessed which species were present in each monolith in 2015.

### Statistics

All statistical analyses were performed with the R statistical package (version 3.4.3; R Development Core Team, 2017). The design was a two-factor split-plot arrangement with monoliths nested within sites (*n* = 6 replicates). The categorical factor irrigation included two levels (irrigation vs. no irrigation). The continuous factor heat included four levels (sites). Two different measures of heat were used as explanatory variables: vapor pressure deficit (VPD) and air temperature (T_air_). In both cases we used the average daytime values during the period of the extremes. These were 0.55 kPa and 14.1°C (Furka), 1.08 kPa and 19.8°C (Oberwald), 1.56 kPa and 24.5°C (Bister) and 1.19 kPa and 23.8°C (Visp), respectively (for further information, see De Boeck et al., 2016). Separate models were fitted for each of these variables because the two are correlated (*r* = 0.95). We consider VPD as the most relevant explanatory variable, as it combines temperature and humidity, two important parameters in assessing heat and drought effects (De Boeck et al., 2011). All response variables were transformed to achieve normal distribution of the residuals and then tested using ANCOVA with VPD or T_air_ as a continuous fixed factor, irrigation treatment as a categorical fixed factor, and monolith (nested within site) as a random factor. Separate analyses were performed for data collected in 2014 and 2015. Nitrogen fixers were not analyzed separately as this group contained only one species (*Trifolium alpinum*), and was therefore lumped together with the other dicots (‘forbs’).

## Results

### Meteorology

**Figure 1:**
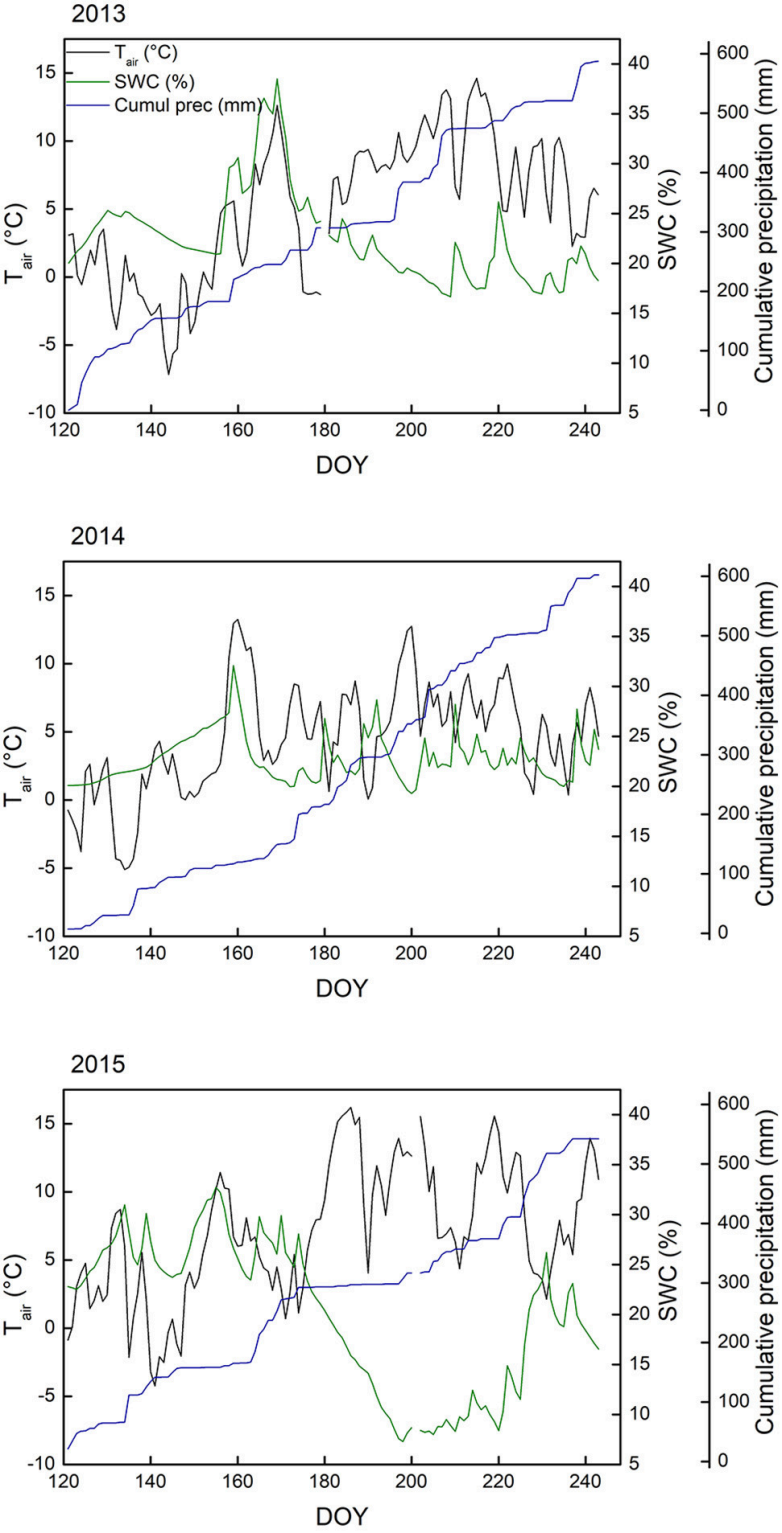
Meteorological data collected at the ALPFOR weather station (2440 m a.s.l., Switzerland), where the alpine grassland in our experiment was located. Depicted are daily means of 2-m air temperature (T_air_), volumetric soil water content (SWC) at 5 cm depth and cumulative precipitation, from May 1st (day of the year, DOY, 121) until August 31st (DOY 243) for 2013, 2014, and 2015.

General environmental conditions varied in several ways during the 3 years this study spans (Table 1, Figure 1). One of the most relevant differences was the date the ground was free of snow, which is highly important regarding the start of the growing season (Körner, 2003). The spring of 2013 was characterized by generally cool conditions, with several late snowfall events. The snowpack was only gone June 19 (DOY 170), whereas snowmelt came markedly earlier in 2014 (DOY 159) and 2015 (DOY 157). While 2013 (after the cool spring) and especially 2015 had warm summers (mean June-August air temperature of 7.1°C and 8.9°C, respectively), 2014 was cooler for the same period (6.0 °C) and also less sunny (−18 and −12% direct radiation compared to 2013 and 2015, respectively). The 2015 (early) summer saw little precipitation, resulting in soil surface drying (Figure 1). Relative air humidity was 74% in 2015, compared to 78% in 2013 and 83% in 2014. It is important to keep in mind that these variables describe the general meteorological conditions, while the microclimate the plants experience deviates from the weather station measurements (see Körner and Hiltbrunner, 2017).

**Table 1:** Overview of measured air temperature and precipitation during the three growing seasons in this study.

	**2013**			**2014**			**2015**		
	**June**	**July**	**August**	**June**	**July**	**August**	**June**	**July**	**August**
T_air_ average (°C)	4.2	9.1	8.0	5.9	6.6	5.5	6.3	11.0	9.2
T_air_ min (°C)	−4.0	2.3	0.8	−1.9	−1.6	−1.6	−1.9	0.3	0.0
T_air_ max (°C)	15.8	16.4	19.4	18.0	16.0	12.4	15.4	19.8	18.2
Precipitation (mm)	105	139	138	107	215	171	136	64	185

*Average, minimum (min), maximum (max) 2-m air temperature (T_air_) and cumulative precipitation per month at the ALPFOR weather station (2440 m a.s.l., Switzerland) are given*.

### Plant and community responses

**Figure 2:**
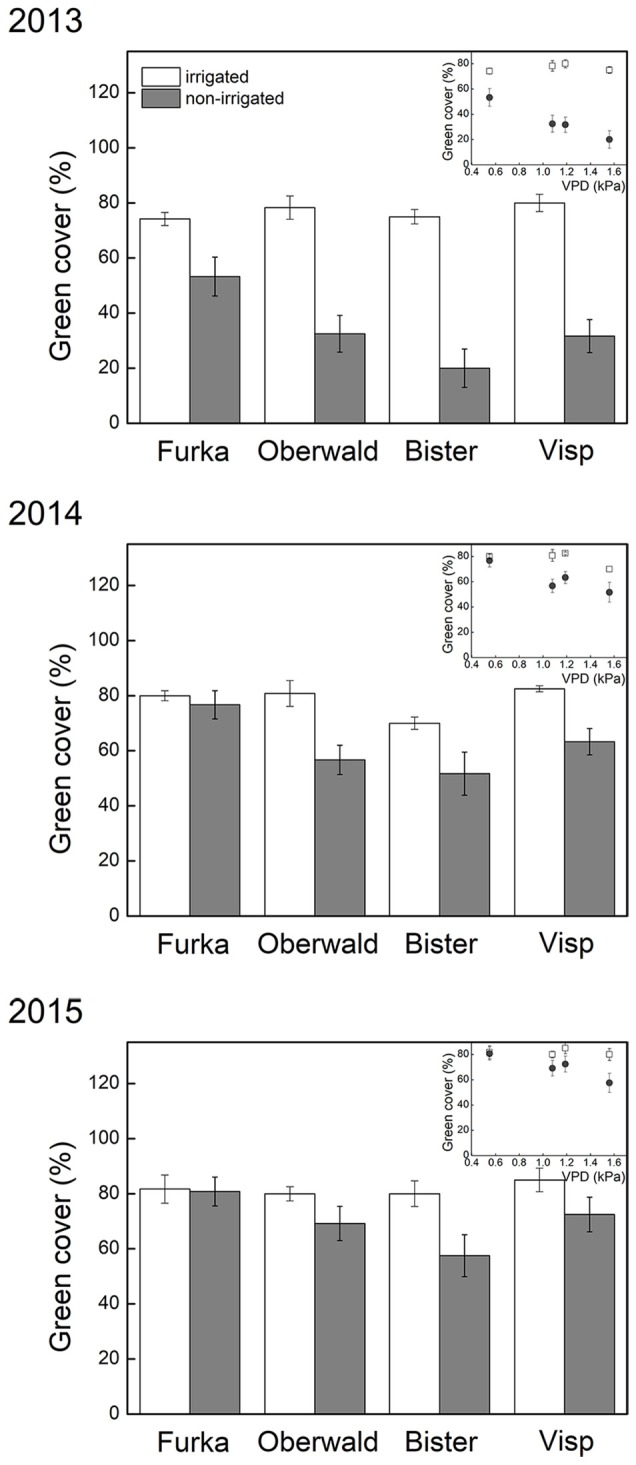
Visual estimates of total green cover (mean ± SE, *n* = 6) taken prior to phytomass harvest, at peak growing season. The X-axis legend refers to the locations of the mesocosms during the 17-day extreme (with or without irrigation) imposed in 2013: Furka (2,440 m a.s.l.), Oberwald (1,390 m a.s.l.), Bister (1,040 m a.s.l.) or Visp (660 m a.s.l.). The inset graph shows the same data of green cover against average daytime vapor pressure deficit (VPD) during the 2013 climate extreme, which was used as explanatory variable in our analyses.

The short-term effects of climate extremes on total green cover detected in 2013 (De Boeck et al., 2016) were still reflected in the observations one and two growing seasons later (Table 2). Communities that had been exposed to heat waves showed very similar green cover (non-significant VPD effect), unless the heat wave had coincided with drought. Indeed, green cover was still significantly reduced 1 and 2 years after drought, and this effect was especially pronounced in monoliths of high-VPD sites, as demonstrated by the marginally significant (2014) and significant (2015) interaction with VPD (Table 2, Figure 2). In other words, 2 years after drought, cover remained suppressed, especially in communities that had been exposed to high atmospheric demand for water (VPD).

**Table 2:** Overview of the outcome of the statistical analyses (ANCOVA) for the response variables 1 and 2 years after the 2013 extreme.

		**2014**						**2015**					
		**VPD**		**Irrig**		**VPD x Irrig**		**VPD**		**Irrig**		**VPD x Irrig**
	**group**	***F*_1, 2_**	***P***	***F*_1, 42_**	***P***	***F*_1, 42_**	***P***	***F*_1, 2_**	***P***	***F*_1, 42_**	***P***	***F*_1, 42_**	***P***
Biomass	all	**43.92**	**0.022**	1.79	0.188	2.67	0.110	0.00	0.975	2.22	0.144	0.22	0.642
	graminoids	**12.11**	**0.074**	1.17	0.286	0.28	0.603	4.36	0.172	0.13	0.721	0.32	0.573
	forbs	**8.81**	**0.097**	**2.87**	**0.098**	**8.40**	**0.006**	0.60	0.519	**10.98**	**0.002**	**4.67**	**0.036**
Necromass	all	0.17	0.723	1.60	0.213	0.17	0.680	0.00	0.985	0.80	0.376	0.02	0.883
Phytomass	all	**51.31**	**0.019**	1.79	0.199	2.59	0.115	0.00	0.988	2.31	0.136	0.20	0.66
Green cover		**10.30**	**0.085**	**23.60**	<**0.001**	**3.4**	**0.072**	6.39	0.127	**9.82**	**0.003**	**4.42**	**0.041**
Species richness	total							2.25	0.272	**4.22**	**0.046**	**4.06**	**0.05**
	graminoids							0.81	0.462	0.00	0.947	0.27	0.609
	forbs							3.41	0.206	**5.54**	**0.023**	**6.20**	**0.017**

*The site-specific average daytime vapor pressure deficit (VPD) during the 2013 extreme was used as a continuous fixed factor, the irrigation treatment in 2013 (Irrig) as a categorical fixed factor, and monolith (nested within site) as a random factor. F-values (including degrees of freedom) and P-values are given with significant (P < 0.05) or marginally significant (P < 0.10) indicated in bold*.

**Figure 3:**
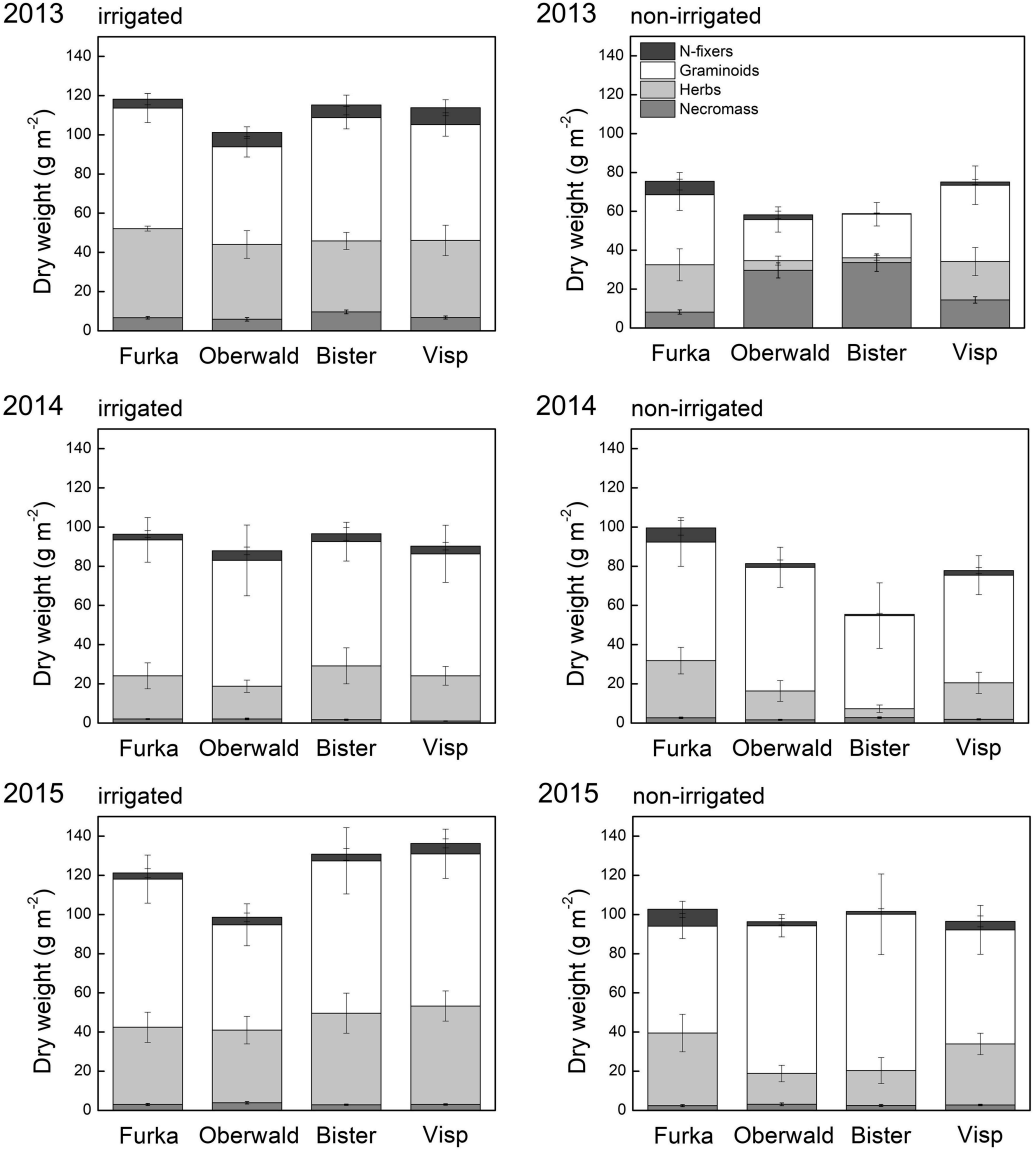
Aboveground phytomass of all monoliths collected around the peak of the growing season, separated per functional group and into living (biomass) and dead (necromass). Error bars depict the standard error (6 monoliths per site and per irrigation treatment). The X-axis legend refers to the locations of the mesocosms during the 17-day extreme (with or without irrigation) imposed in 2013: Furka (2,440 m a.s.l.), Oberwald (1,390 m a.s.l.), Bister (1,040 m a.s.l.) or Visp (660 m a.s.l.).

Contrary to green cover, biomass and phytomass at the community scale had rebounded to control levels by 2015 (no VPD or irrigation effect anymore in 2015, cf. Figure 3). However, there was a trend for drought-suppressed forb biomass in 2014, an effect that had become significant in 2015. Furthermore, we detected a significant interaction between drought and VPD for this group, indicating that the negative drought impact on forb biomass the years after the extreme increased with VPD.

**Figure 4:**
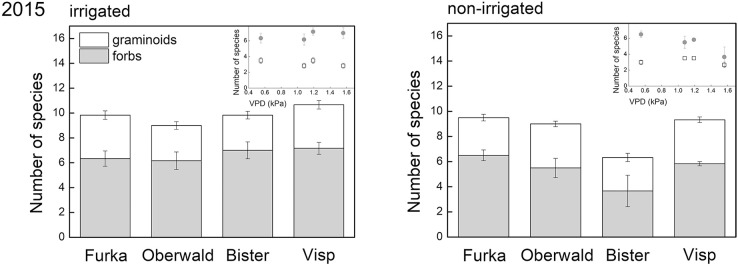
Species richness (per functional group) recorded during the 2015 growing season. The X-axis legend refers to the locations of the mesocosms during the 17-day extreme (with or without irrigation) imposed in 2013: Furka (2,440 m a.s.l.), Oberwald (1,390 m a.s.l.), Bister (1,040 m a.s.l.) or Visp (660 m a.s.l.). The inset graph shows the same data of species richness against average daytime vapor pressure deficit (VPD) during the 2013 climate extreme, which was used as explanatory variable in our analyses.

Although forbs made up less than half of the community biomass in 2015 (controls), more forb species were present (11 in total) than graminoid species (4 in total) 2 years after the 2013 extreme. Both total species richness and species richness of forbs were significantly reduced by drought, especially in monoliths of high-VPD sites (significant interaction), whereas species richness of graminoids was unaffected by the different treatments (Figure 4). These effects mirror those found for the biomass of both groups (Figure 3).

## Discussion

Temperatures in mountain regions are generally rising faster than the global mean, which also increases the probability for exceptionally warm periods (Gobiet et al., 2014). At the same time, changes in precipitation patterns are expected, with more frequent droughts a distinct possibility in the Alps (Heinrich et al., 2014). We here studied if heat waves and/or drought in alpine grassland would lead to discernible legacy effects regarding productivity (here: above-ground biomass formation), green plant cover and species composition the years after the event, and whether these were closely linked to the immediate impacts. The short-term responses to heat waves of varying intensity were subdued and insignificant (De Boeck et al., 2016). We speculated that thresholds for heat stress had not been exceeded because tissue temperatures were moderated via latent heat transfer (transpiration; Larcher, 2003; De Boeck et al., 2011), with stomatal conductance not being affected by warming as such in our experiment (De Boeck et al., 2016). Moreover, alpine plants do experience relatively high tissue temperatures also in their native environment, as a result of high radiation and atmospheric decoupling (Körner, 2003; Neuner and Buchner, 2012; Dietrich and Körner, 2014). The initial lack of responses to heat in the absence of drought was also observed the following growing seasons. Both green cover and biomass did not significantly differ from control in the years after the imposed heat wave. Likewise, no significant changes in species richness or the balance between the functional groups present were observed. This also implies that potential indirect effects from the heat wave, for example through warming-induced changes in nutrient availability (Schmidt et al., 2002; Cross et al., 2015) or shifts in allocation to storage organs (Xu et al., 2016), were minor or absent.

Whereas these alpine grasslands were stable in both the short and longer term when faced with heat waves under non-limiting soil water, this changed when drought co-occurred. Drying soils and high VPD led to closure of stomata and loss of transpirational cooling, likely resulting not only in drought stress, but also heat stress (cf. Buchner et al., 2017) which in turn caused significant decreases in green cover and biomass, and increases in necromass (De Boeck et al., 2016). Our measurements demonstrate that trends observed on the short-term persisted the following 2 years, especially regarding green cover (Figures 2, 3). This implies that the initially observed senescence was not, or not merely, a response that avoided further dehydration so that cell integrity in meristems could be maintained, but an indicator of vulnerability to the imposed stress (Zwicke et al., 2015; Stampfli et al., 2018), confirming hypothesis 1. The significant interaction of green cover and VPD (i.e., site) reflects the complete recovery for the communities that experienced only drought (“Furka”), while cover continued to be below reference (control) levels elsewhere, especially in the communities that had been exposed to the hottest and driest conditions (“Bister”). Reduction in cover may increase the exposure of soils to heavy precipitation events (Zuazo and Pleguezuelo, 2009), in turn affecting nutrient availability and soil stability. Here, the relatively large root biomass in alpine grassland (Körner, 2003) would decrease the vulnerability to such impacts, however. Productive temperate grasslands tend to bounce back to prior growth levels rapidly, often within weeks of the extreme (Dreesen et al., 2014; Zwicke et al., 2015). Fast recovery is stimulated in such grasslands by generally high available soil nutrients, especially immediately following a drought (the Birch effect, cf. Birch, 1958; Ingrisch et al., 2018). However, rapid recovery was not observed in our alpine grassland. It is possible that low nutrient availability played a role (Gessler et al., 2017), and it is likely that phenological constraints severely limited potential (re)growth later in the (2013) growing season (Körner and Pelaez Menendez-Riedl, 1989).

The slow (literal) recovery and lack of new species observed in communities with the highest mortality suggests that gaps tended to close through encroachment rather than recruitment from seed. The alpine grassland in our study consists mostly of long-lived individuals that often reproduce clonally (De Witte et al., 2012) and lacks annual species that can quickly colonize open niches. This implies that compensation for negative impacts of extremes through recruitment, as observed in communities where dominant species have a short life cycle or produce abundant seedlings (Aragón et al., 2010; Lloret et al., 2012), is limited here. Such fast compensation is more important in temperate grasslands, where the longer growing season presents a longer window of opportunity for recovery (De Boeck et al., 2011). Moreover, the sometimes harsh microclimatic environment in gaps in high alpine grassland (Lembrechts et al., 2015) makes seedling establishment more difficult, and thus a limiting factor in these systems. It should be noted that whereas our imposed extremes affected only the monoliths and not the landscape (an inherent problem of manipulation experiments, see De Boeck et al., 2015), a natural, landscape-wide hot drought could affect propagule rain (Zeiter et al., 2006; Ertl, 2013), with implications for recruitment from seed.

In spite of slow restoration of green cover after extremes, the productivity of the alpine grassland in our study did bounce back faster. Cover and biomass are not necessarily correlated, as the latter also depends on plant height, canopy structure and community composition (Axmanová et al., 2012; Jiang et al., 2017). In our case, the graminoid fraction in the vegetation, with its more erect growth form, likely compensated for the reduced share of forbs compared to communities not exposed to drought, which led to restored community productivity in spite of lower green cover. Both the extremes and the changes in community composition could have altered root biomass and vertical distribution of roots (Liu et al., 2018), but were not studied here.

The relative success of graminoids was more pronounced in treatments where the remaining (living) biomass was lowest directly after the extreme event and necromass highest (Oberwald and Bister, Figure 3). The damage to forbs had been significantly higher than to graminoids under those hot and dry conditions, possibly resulting from leaf size (larger) and orientation (more perpendicular to midday sun) and ensuing higher tissue temperatures (Dietrich and Körner, 2014; De Boeck et al., 2016). In addition, the forb species present in our study had a significantly higher specific leaf area than the graminoids (abundance-weighted means, data not shown). Specific leaf area has been linked to water and nutrient economy, suggesting that higher values are more common for fast-growing (resource acquisitive) species, while lower values are found more often in slow-growing (resource conservative) species (Reich, 2014). Indeed, the dominant graminoids in this alpine grassland, *Carex curvula* and *Nardus stricta*, are known to have high leaf longevity, which is inversely related with specific leaf area (Körner, 2003). This conservative strategy has been associated with increased resistance to drought compared with the resource acquisitive strategy (Lepš et al., 1982; Grime et al., 2000) and could thus help explain the higher survival of the graminoid fraction in our communities in the face of hot droughts. An increased proportion of graminoids vs. forbs after drought has been observed in other grassland studies in the Alps (Ingrisch et al., 2018; Stampfli et al., 2018), but also elsewhere (Mulhouse et al., 2017). Of special note is that our experimental findings were corroborated by the observational study of Cremonese et al. (2017), who found that a pronounced natural hot drought in the Italian Alps impacted forbs (of which several species were identical to our study) significantly more than grasses. Forbs showed some signs of recovery in absolute terms the years after our imposed extreme, but remained suppressed compared to controls with their share around 20% (compared to c. 35% in controls). Other than forbs making up a reduced proportion of community biomass, forb species richness had also significantly declined. The ability of graminoids to rapidly resprout from basal meristems and “grab” the available nitrogen (Volaire et al., 2014) after the mortality of especially forbs may have helped to consolidate their increased share and increase community production back to control levels two growing seasons after the extremes.

Changes in the balance between functional groups can have profound implications for ecosystem functioning. In our case, above-ground biomass production was restored, but other functions may have been altered, such as feed and pollinator value. Carnicer et al. (2011) reported that herbivory, as well as pest or pathogen density can be reduced after mortality of a dominant plant species, although we have no indications for that here. Nutrient cycling may be affected by compositional changes cascading to soil communities via altered inputs (Wardle et al., 2004; De Deyn and Van der Putten, 2005). Nevertheless, the recovery of biomass production seems to indicate that at least initially, any changes in nutrient cycling did not play a major role in this alpine grassland. Another potential consequence of changes in community composition is an altered response to new extremes. As graminoids resisted the original extreme events best, new episodes of heat and drought combined may have less effect in such “pre-adapted” communities and could thus prolong graminoid dominance. In the future, climate extremes are projected to not only become more intense, but also more frequent. Such changes in the weather regime may thus perpetuate shifts arising from single extreme events (cf. Kreyling et al., 2011). This is an important aspect of the impacts climate change may have in these alpine grasslands that should be considered next to the effects of changes in the mean climate, such as species migration and altered phenology, including the ensuing changes in competitive interactions (Alexander et al., 2016; Rumpf et al., 2018; but see Scherrer and Koerner, 2010 on the importance of microtopography).

In conclusion, even though the immediate responses to heat extremes with or without drought seemed similar to the ones repeatedly observed in lowland grassland (De Boeck et al., 2011; Dreesen et al., 2014), alpine grassland showed longer legacy effects (cf. Wu et al., 2018). The short growing season and difficult seedling establishment constrained recovery, at least from the annual perspective (see Körner, 2003 and Körner, 2013 regarding annual versus growing season points of view). Return to normal functioning regarding aboveground biomass production took 2 years, though green cover was still suppressed in some treatments, while the prior (“normal”) state was not reached from the perspective of diversity and community structure. Our data suggest that these alpine systems may become relatively enriched with graminoid species following hot droughts while forbs would decline, both in terms of biomass and species richness. While the ecosystem should return back to its prior state slowly, a new climate regime with more frequent extreme events could perpetuate the shift in community composition we observed. The relative enrichment with resource conservative graminoids may improve the system's resistance in the face of new climate extremes, while some of its functioning is bound to be different. Slow recovery after mortality hints at the importance of initial resistance to a climate extreme in shaping these alpine grasslands the years after the event.

## Author contributions

HD designed the study. HD and MV performed the measurements, with help from EH, SB and MZ. MZ analyzed the data. HD and MZ wrote the first draft. All authors contributed substantially to revisions and gave final approval for publication.

### Conflict of interest statement

The authors declare that the research was conducted in the absence of any commercial or financial relationships that could be construed as a potential conflict of interest.
